# Unicentric Castleman's Disease Arising from an Intrapulmonary Lymph Node

**DOI:** 10.1155/2013/289089

**Published:** 2013-06-10

**Authors:** Hideki Ota, Hideki Kawai, Tsubasa Matsuo

**Affiliations:** Department of Thoracic Surgery, Akita Red Cross Hospital, Akita 010-1495, Japan

## Abstract

Castleman's disease is an uncommon lymphoproliferative disorder of unknown etiology, most often involving the mediastinum. It has 2 distinct clinical forms: unicentric and multicentric. Unicentric Castleman's disease arising from an intrapulmonary lymph node is rare, and establishing a preoperative diagnosis of this disease is very difficult mainly due to a lack of specific imaging features. We report a case of intrapulmonary unicentric Castleman's disease in an asymptomatic 19-year-old male patient who was accurately diagnosed by preoperative computed tomography (CT). The mass was incidentally found on a routine chest X-ray. A subsequent dynamic CT showed a well-defined, hypervascular, soft-tissue mass with small calcifications located in the perihilar area of the right lower lung. Three-dimensional CT (3D-CT) angiography indicated that the mass was receiving its blood supply through a vascular network at its surface that originated from 2 right bronchial arteries. The clinical history and CT findings were consistent with a diagnosis of unicentric Castleman's disease, and we safely and successfully removed the tumor via video-assisted thoracoscopic surgical lobectomy. This case shows that the imaging characteristics of these rare tumors on contrast-enhanced CT combined with 3D-CT angiography can be helpful in reliably establishing a correct preoperative diagnosis.

## 1. Background

Castleman's disease, also known as giant lymph node hyperplasia and angiofollicular lymphoid hyperplasia, is an uncommon lymphoproliferative disorder of unknown etiology that was first reported by Castleman and Towne in 1954 [1]. Castleman's disease is clinically classified into 2 distinct presentations, unicentric (localized) and multicentric (systemic) [2, 3]. Most cases of unicentric Castleman's disease are found in the mediastinum and abdomen [4, 5], but occasionally it will involve intrapulmonary lymph nodes [5–10]. Complete surgical resection is the standard treatment of unicentric Castleman's disease, including cases arising from intrapulmonary lymph nodes. However, establishing a preoperative diagnosis can be very challenging because there are few distinguishing imaging characteristics. Here, we present a case of intrapulmonary unicentric Castleman's disease in a patient who underwent curative surgical resection, and we review the pertinent literature.

## 2. Case Presentation

A 19-year-old, nonsmoking, male patient was referred to our department for further evaluation of an abnormal shadow on a routine chest radiograph. The patient was asymptomatic with no past medical history, and physical examination findings were unremarkable. His hematological and biochemical parameters were within normal limits. 

**Figure 1:**
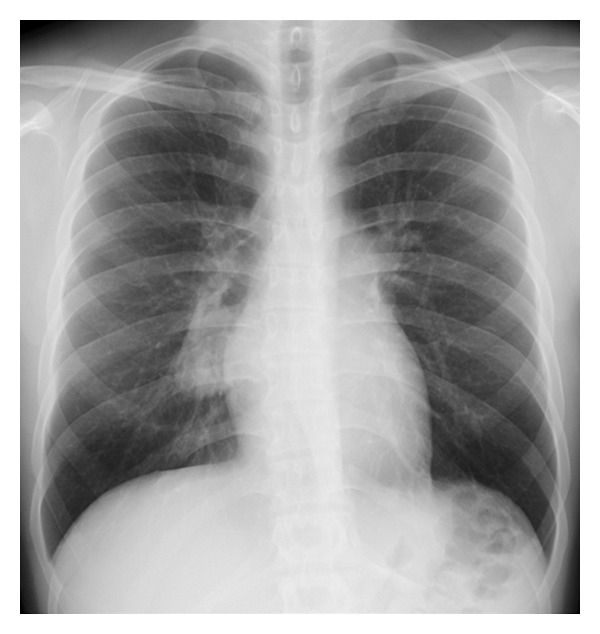
Chest radiograph shows a mass lesion located in the right infrahilar region.

**Figure 2:**
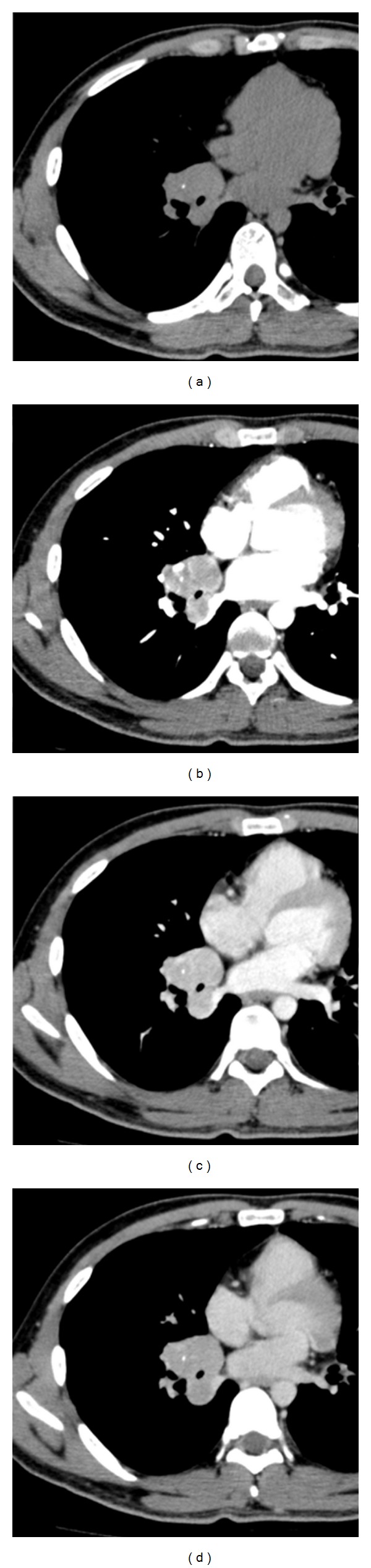
Dynamic CT scans of the chest, in plane (a), arterial (b), portal in-flow (c), and equilibrium (d), show a well-defined, lobulated, soft-tissue mass with small calcifications in the right lower lobe. The periphery of the mass was strongly enhanced in the arterial phase, and the parenchyma of the mass was heterogeneously enhanced in the portal in-flow to equilibrium phase.

**Figure 3:**
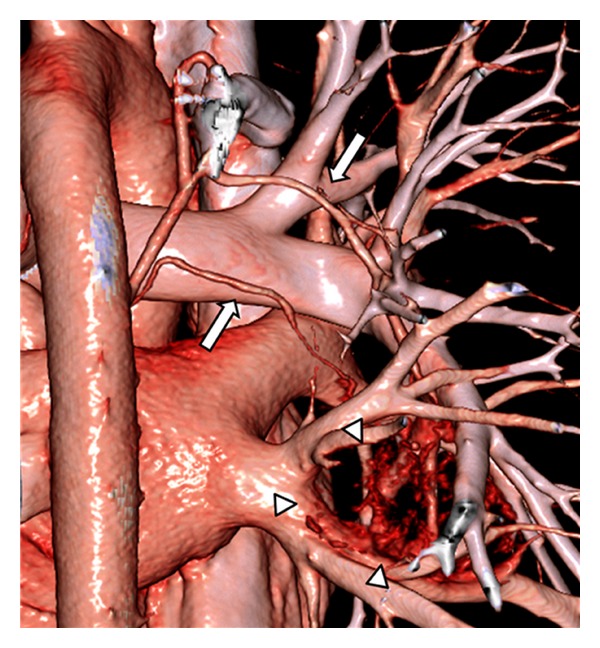
3D-CT angiography demonstrated the enlarged feeding vessels at the surface of the mass (arrowheads) originating from 2 bronchial arteries (arrows).

Plain chest radiograph showed a tumor mass in the right infrahilar region (Figure 1). Computed tomography (CT) further described a well-defined lobulated mass of soft-tissue density with calcifications, measuring 5.0 × 4.8 cm in the axial plane, and confirmed its location in the right lower lobe (Figure 2) around the intermediate and basal bronchi of the right lung, where it was compressing contiguous vascular and bronchial structures. No other enlarged lymph nodes were seen. Dynamic CT showed contrast enhancement beginning in the periphery of the homogenous mass and becoming diffuse. Finally, three-dimensional CT (3D-CT) angiography revealed that the mass was receiving a rich blood supply from a vascular network at its surface originating from 2 right bronchial arteries (Figure 3). On magnetic resonance imaging (MRI), the tumor was isointense to muscle on T1-weighted imaging and hyperintense on T2-weighted images with heterogeneous enhancement in dynamic contrast sequences. Endoscopic bronchial ultrasound confirmed increased vascularity at the surface of the tumor, and bronchoscopy did not detect any abnormalities.

**Figure 4:**
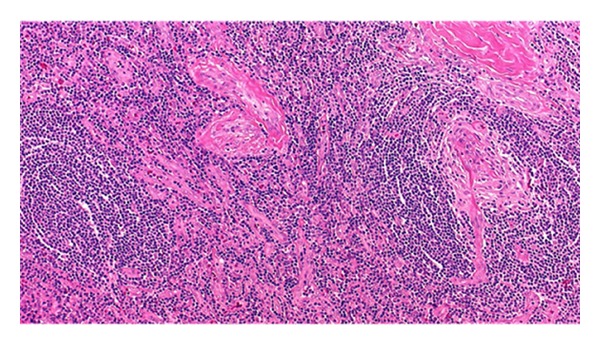
Histology shows the typical appearance of hyaline vascular type Castleman's disease.

The patient's history and the findings of hypervascularity with small calcifications in the intrapulmonary soft-tissue mass supported a diagnosis of unicentric Castleman's disease. We performed video-assisted thoracoscopic surgery and found an elastic hard tumor that was tightly adhered to the intermediate-to-common basal bronchi. The tumor had abundant arterial supply from the 2 right bronchial arteries. The tumor was totally removed via a right middle-lower lobectomy. Macroscopically, the cut surface of the lesion was yellow-white, well-circumscribed, and encapsulated and measured 5.2 × 5.0 × 3.8 cm (Figure 4). Histologically, the lesion showed proliferation of lymphoid follicles with hyalinized vessels in the center of the follicles, along with concentric layering of lymphocytes in the periphery, creating an onion-skin appearance. These findings confirmed the diagnosis of hyaline vascular type unicentric intrapulmonary Castleman's disease. The patient had an uneventful postoperative course and was discharged 4 days after surgery. As of this report, he had remained well with no recurrence for 8 months.

## 3. Discussion

Unicentric Castleman's disease is a localized form of hypervascular lymphoid hyperplasia. In cases of intrapulmonary disease, it arises from a lobar or segmental lymph node of the lung [5–10]. Establishment of a conclusive diagnosis without a tissue specimen is very difficult because the tumors lack reliably distinguishing imaging characteristics. In the present case, asymptomatic intrapulmonary unicentric Castleman's disease in a 19-year-old male patient was discovered incidentally on a routine chest X-ray (Figure 1) and was further characterized on contrast-enhanced CT scan and 3D-CT angiograms as a hypervascular intrapulmonary soft-tissue mass containing small calcifications and supplied by multiple feeding vessels from 2 bronchial arteries. These imaging features played an important role in making a correct presumptive diagnosis and in guiding careful surgical planning to minimize risk of hemorrhage.

The unicentric and multicentric forms of Castleman's disease have 3 histologic subtypes: hyaline vascular, plasma cell, and mixed. Unicentric Castleman's disease is usually of the hyaline vascular type and multicentric disease of the plasma cell or mixed type. Unicentric Castleman's disease presents as a slow-growing mass that is usually found in asymptomatic young adults, without gender or race predominance [2], and it is known to follow a relatively benign clinical course until and after complete surgical resection [11, 12].

The most common site of involvement of unicentric Castleman's disease is the mediastinum (70%) [4], but it may occur anywhere in the lymphoid chain [5]. We found 6 previously reported cases of unicentric Castleman's disease of intrapulmonary lymph nodes in the English literature to date [5–10]. Including the present case, the patients are 4 males and 3 females, ranging in age from 19 to 54 years (median 27 years). Three patients have presented with symptoms resulting from compression of the adjacent bronchus, 2 with cough and 1 with back pain. The tumors range in size from 3.5 to 7.5 cm, with a mean of 5.0 cm. Three were located in the right lung, 4 in the left lung. Histologically, all of the tumors were hyaline vascular type. Five patients were treated by lobectomy and 2 patients were treated by tumor dissection. None of the patients had recurrence during reported followup.

The difficulty of diagnosis of intrapulmonary unicentric Castleman's disease without a tissue specimen is also a concern with these hypervascular masses because of the risk of massive hemorrhage during biopsy by transbronchial needle aspiration or during excision by thoracotomy. Nontissue diagnosis is very difficult mainly because of the lack of reliable specific findings on most imaging studies [5–10]. It is important to establish typical imaging characteristics of this rare disease in order to avoid unnecessary surgical interventions and complications. As described, CT scan generally shows a solid homogeneous soft-tissue mass with clear contrast enhancement, which begins in the periphery and becomes diffuse [13–16]. In some cases, the degree of enhancement of intrapulmonary lesions is less than that of those in other locations [6]. Larger lesions (>5 cm) may demonstrate a heterogeneous appearance associated with central necrosis or degeneration [14]. In nearly 30% of cases, the mass is accompanied by stellate microcalcifications [16]. Similar to findings described in other reports [16, 17], our patient had a well-defined mass that was isointense to muscle on T1-weighted MRI images and hyperintense on the T2-weighted images. The 3D-CT angiography finding of a hypervascular mass with numerous enlarged feeding vessels has also been reported in unicentric Castleman's disease, along with a homogeneous capillary blush corresponding to capillary proliferation, which is typically seen in the hyaline vascular types of these tumors. While all of these imaging findings are nonspecific, the presence of microcalcifications and hypervascularity combined with other clinical characteristics, such as young age and otherwise negative medical history, can indicate a strong possibility of unicentric Castleman's disease [18], with a differential diagnosis including carcinoid tumor and lymphoma, which will manifest as a perihilar mass with a close anatomic relationship to the lobar or segmental bronchi, similar to the lesion in the present case. Carcinoid is usually confirmed by bronchoscopy, and lymphoma is generally not enhanced by contrast material [19].

The imaging characteristics found in our patient were almost the same as those described previously, which enabled us to establish a correct preoperative diagnosis, despite the unusual tumor location of involvement, and to our knowledge, this is the first report to describe the 3D-CT angiographic features of a unicentric Castleman's disease arising from an intrapulmonary lymph node. Although 3D-CT angiography did not clearly demonstrate a homogeneous capillary blush in our case, we believe that, combined with the other findings described herein, it did provide a less invasive, more efficient, and safer alternative to conventional angiography in formulating a preoperative diagnosis and an appropriate surgical plan. We found that the angiographic features of 3D-CT angiography were useful for diagnosing and successfully treating this rare case of intrapulmonary unicentric Castleman's disease. 

## 4. Conclusion

We report a rare case of unicentric Castleman's disease arising from an intrapulmonary lymph node. The findings of hypervascularity at the surface of a well-delineated homogenous mass accompanied by small calcifications and located in the perihilar area of the lung proved to be reliable imaging characteristics in guiding our diagnosis of this rare tumor. Castleman's disease should be part of the differential diagnosis when an intrapulmonary mass with the features described in this report is found in a young patient. Complete surgical resection guided by an accurate preoperative diagnosis will achieve the best results.

## Abbreviations

CT: Computed tomography
3D-CT: Three-dimensional CT
MRI: Magnetic resonance imaging.
